# Response of Polish Psychiatric Patients to the Russian Invasion of Ukraine in February 2022―Predictive Role of Risk Perception and Temperamental Traits

**DOI:** 10.3390/ijerph20010325

**Published:** 2022-12-25

**Authors:** Magdalena Nowicka, Ewa Jarczewska-Gerc, Magdalena Marszal-Wisniewska

**Affiliations:** 1 Institute of Psychology, University of Social Sciences and Humanities, Chodakowska 19/31, 03-815 Warsaw, Poland; 2 Faculty of Psychology, University of Social Sciences and Humanities, Chodakowska 19/31, 03-815 Warsaw, Poland

**Keywords:** war, schizophrenia, depression, psychopathological symptoms, risk perception, temperament

## Abstract

This study examined the impact of the Russian invasion of Ukraine in February 2022 on Polish patients with depression and schizophrenia. It has been hypothesized that possible changes in symptoms may be predicted by the subjective risk perception related with the Russo-Ukraine War (RUW) as well as by temperamental traits. The study was conducted with 80 patients with schizophrenia or depression. A questionnaire measuring sociodemographic characteristics, perception of risk, temperamental characteristics, and symptoms of depression and schizophrenia were used as research tools. Symptom intensity was compared with the average symptom intensity calculated on the basis of archival symptom measurements from the three consecutive months preceding the outbreak of the RUW. Predictors of change in symptoms were also analyzed, taking into account sociodemographic variables, the level of risk perception, and temperamental traits. The results indicate the specific patterns of reactions to war danger for patients with different types of psychiatric diagnosis. Depressed patients reacted with an increase in seven symptoms related to unworthiness and/or guilt, lack of interest, and/or pleasure and pessimism. The response of schizophrenic patients was related only with an increase in positive symptoms. This study highlights the need to provide more support to psychiatric patients in acute emergencies.

## 1. Introduction

### 1.1. Background

On 24 February 2022, Russia attacked Ukraine, taking the next step in the escalation of a long-term conflict. The Russian invasion of Ukraine caused a great sense of danger in all Europe. Moreover, this conflict brought into the question the adequacy of current world peace policy. The analyses of the EU’s attitude to the Russo-Ukraine War (RUW) showed deep divide between members of the European Union [1].

War is an intense, traumatic experience, with the high risk of psychical and physical health complications [2,3,4]. So far, RUW have caused enormous psychological consequences for Ukrainian people [5], as well as for other European citizens [6]. As two neighboring countries, Poland and Ukraine have a long history of friendship between common people as well as two Slavic nations. Due to the strong relations, the outbreak of RUW was extremely traumatic for Polish citizens. It should also be noted that Poles have welcomed more Ukrainian refugees than any other country in Europe. In the first month of the war, hundreds of thousands of Polish families opened their homes to Ukrainian people and heard first-hand dramatic relations about the RUW. In the CATI survey conducted in May and June 2022 by the University of Warsaw [7], 70% of interviewed Polish citizens declared that the war in Ukraine has raised real and strong concerns about their future. Data collected by The Public Opinion Research Center (CBOS) in June and July 2022 [8] suggests that the majority of Poles are indeed afraid of a Russian armed attack on Poland. Analogical observations result from the study of the population of Polish students [9]. The fear of Poles seems to be intensified by numerous historical experiences (e.g., the Polish-Bolshevik War of 1919, Russian occupation of Poland during World War II). Moreover, it is exacerbated by current events taking place directly in Poland. This article was written a few days after an unexpected explosion in a small village near the Poland-Ukraine border; two people died as a result. This event caused a real fear of Russian attacks on Poland.

So far, a considerable body of research has demonstrated increased psychopathology in both soldiers and civilian casualties of war [10]. Military conflict induces strong psychological reactions such as panic attacks or shell shock, as well as more complex syndromes such as Posttraumatic Stress Disorder (PTSD) [11,12,13,14]. The various aspects of war induced psychological effects were studied in the population of adults [15], adolescents [16,17] and children [18,19]. It can be concluded that an important and urgent task for psychologists today is to study the effects of war on populations of special needs. Psychiatric patients are considered weaker and less resilient than others and thus constitute a high risk group for further mental breakdown in the face of war danger [14]. So far, their response to war stress has gained limited scientific attention. Even if such studies are published, they suffer from methodological shortcomings [only observational or retrospective data] and usually do not take into account predictive factors such as the level of risk perception or more stable individual traits (e.g., personality or temperamental traits) [4,16,17,18,19].

Scientific literature proposes various models to explain the possible reaction of psychiatric population to high adversity and extreme stress [20]). Only three of them have been positively, albeit narrowly, verified in the context of war conflict.

The first of the models, based on the concept of important life changes [20,21,22] states that an extremely stressful situation will aggravate illness or unmask a latent disease. A mentally ill person will respond to extreme stressors with regression and/or symptom aggravation. The hypothesis was confirmed in the condition of a military conflict during the assassination of Indira Gandhi in India (patients with various diagnoses―anxiety disorders, schizophrenia) [23]. Partial verification was also found in the study of schizophrenia patients during the 1991 Gulf War (only anxiety symptoms aggravation) [16].

The second model assumes that mental illness may paradoxically serve as a buffer against extremely strong stressful situation [20]. Therefore, war may have no significant effects on individuals with psychiatric diagnosis or may produce only slight changes. This hypothesis seems to be more possible in populations of psychotic patients who invest cognitive and emotional energy especially in their internal world. Such thesis was partly supported by Bromet et al. [21] who found that psychiatric patients exposed to the Three Mile Island Accident did not differ in anxiety, depression and psychotic symptoms intensity from those who were not exposed to this stressor. Analogical results were also obtained by Grisaru et al. [24] and Bendor et al. [16].

The third model maintains that war may distract mentally ill people from his/her inner turmoil and force the individual to face the demands of reality [4,20]. The process can result in improved functioning of the individual and reduced manifestation of symptoms. As in the case of the two models mentioned above, this hypothesis has been confirmed in limited number of empirical research. The data obtained by Weil [4] suggested a process of the reduction of symptoms in the population of psychiatric patients during the eighth week of the Yom Kippur War 1973.

### 1.2. Aim

In the study described in this publication, it was decided to investigate the impact of RUW 2022 on Polish patients with depression and schizophrenia. We hypothesized that possible symptom changes may be predicted by subjective risk perception related with RUW, as well as by temperamental traits distinguished in Regulative Theory of Temperament (RTT) [25] that defines seven traits, of which emotional reactivity seems to be the most strongly related to different symptoms of psychopathology [26,27,28]. In addition, research based on RTT has already generated useful information on the predictive role of temperament in the dominant coping style and in response of people in extremely stressful situations [25,28,29].

## 2. Materials and Methods

### 2.1. Sample

The test group consisted of 80 psychiatric patients from an outpatient clinic in central Poland (a small city of thirty thousand inhabitants), including:

- schizophrenia patients, *N* = 40 (*M_age_* = 29.61, *SD* = 10.12);
- depressed patients, *N* = 40 (*M*_age_ = 31.12; *SD* = 8.16).

Both groups begun psychiatric treatment approximately 64 months before the RUW and continued after the outbreak of the war (*M* = 64.25; *SD* = 5.11). The criteria for inclusion in the group were: psychiatric diagnosis of one of above-mentioned diseases, no ongoing process of individual/group psychotherapy, type of psychiatric treatment, stability of mental symptoms during the three months before the outbreak of RUW [researchers assessed symptoms stability based on archival psychiatric symptom measurements (data from medical records of an outpatient clinics in central Poland) (BDI-II and PANSS) over three consecutive month prior to the outbreak of RUW; no significant differences in one-way ANOVA for repeated measures], and willingness to participate in the study. All patients with schizophrenia were taking atypical antipsychotics drugs (Olanzapine) and had no depressive symptoms in the last year of treatment (criteria presented by Gozdzik-Zelazny, A. et al. [30]). Depressed patients were taking typical selective serotonin reuptake inhibitors (Citalopram, Escitalopram, Fluoxetine, Sertraline) and showed no psychotic symptoms. All respondents denied using drugs (during the 12 months before study). They also did not report neurological or somatic disorders.

### 2.2. Procedure

The study used two main symptom measures: Beck Depression Inventory-II (BDI-II) for patients with depression [31,32], and Positive and Negative Syndrome Scale (PANSS) for patients with schizophrenia [33]. Based on these methods, the intensity of symptoms was assessed two weeks after 24 February 2022 (main measurement). In the next step, the obtained results were compared with the average symptoms intensity calculated on the basis of archival measurement of symptoms from three consecutive months before the outbreak of RUW (base measurement―November 2021, December 2021, and January 2022).

During the assessment in February 2022 each of patients responded additionally to the survey referring to sociodemographic data and risk perception as well as the Formal Characteristics of Behavior―Temperament Inventory (revised version) (FCB-TI(R)) [34].

The research project was approved by the Research Ethics Committee at the Faculty of Psychology, SWPS University in Warsaw.

### 2.3. Measures

In the group of schizophrenia patients, psychiatric symptomatology was measured using the PANSS scale, which consists of 30 symptoms including a range of positive (e.g., delusions and hallucinations) and negative symptoms (e.g., blunted affect, poor rapport) as well as symptoms of general psychopathology, such as tension, somatic concern, and anxiety [33]. The presence and severity of each symptom was rated by a well-trained observer on a 7-point Likert scale (1―no symptoms to 7―extreme). Based on the above scoring method, the mean scores for the Positive, Negative, and General Subscale were calculated. Strong psychometric properties for the scale (reliability, validity, and sensitivity) have been demonstrated in many subsequent studies (for summary see [35]). Ratings of symptoms in the base and main measurements were assigned during individual evaluation meetings by psychologists with a doctorate or master’s degree and involved the use of data from patient reports, caregiver reports, and clinical observations.

In the group of depressed patients, the intensity of 21 depressive symptoms was measured during base and main measurements using BDI-II [31]. In this form each symptom is rated by patients on a four-point scale from 0 to 3, and the total score can range from 0 to 63. The Polish version of this scale is reliable and has satisfactory accuracy and sensitivity [32].

In all cases, temperamental traits were measured using FCB-TI(R) [34]. This is a 100-item self-report scale (with 1 not at all―4 very much answer form), measuring seven temperamental traits: Briskness (BR), Perseveration (PE), Sensory Sensitivity (SS), Emotional reactivity (ER), Endurance (EN), Activity (AC), and Rhythmicity (RT). The inventory has good psychometric properties with reliability (Cronbach’s α) ranging from 0.73 to 0.88 and high theoretical validity.

To assess the perception of RUW-related risk, a pre-validated short questionnaire containing the following 4 items was used [36]:

- Health Risk (HR): patients had to express the negative effect of RUW on their own psychical health (0―not at all, 10―very much);
- Psychological Risk (PR): patients had to express how the RUW would negatively affect their well-being (emotional stability, social relations and spirituality) in the future (0―not at all, 10―very much);
- Global War Risk Perception (GWR): patients had to express the likelihood of war occurring in countries neighboring to Ukraine, including in Poland (0―not at all, 10―very much);
- Institutional-Economy Risk Perception (I-ER): patients had to indicate the possible negative role of RUW for world peace and the financial crisis (0―not at all, 10―very much).

The above questionnaire of risk perception also collected some demographic data: marital status, number of children, years of education and work experience.

### 2.4. Statistical Analyses

All variables analyzed in this study were firstly checked for normality of distribution using the Kolmogorov–Smirnov test. Non-normally distributed quantitative data were presented as medians and interquartile ranges, normally disturbed data as means and standard deviations, and categorical variables as counts and percentages. Non-normally distributed variables in two group of patients were compared using the Kruskal–Wallis test, and normally distributed―using a t-test. A Chi-squared test was used to compare categorical variables. Multiple univariate regression models (with Bonfferoni correction) were used to determine whether the indexes of symptom change in each patient group could be predicted by various exploratory variables (sociodemographic variables, risk perception, and temperamental traits). The program used for these analyses was IBM SPSS Statistics 21.0

## 3. Results

Comparing both groups of patients in terms of sociodemographic variables, significant differences were found only within two variables. Depressed individuals declared more years of education and a longer period of employment than schizophrenia patients (all *p* < 0,05). These results are quite obvious from the perspective of early onset of schizophrenia and significant impairment of social functioning in population of schizophrenics. Results are shown in Table 1.

Differences between the two groups of patients in the perception of risk associated with RUW and the intensity of temperamental traits are presented in Table 2.

Regarding temperamental traits, depressed patients were more emotionally reactive and less active than schizophrenia patients (all *p* < 0.05). Analysis of the risk-related variables showed that patients with depression perceive a greater risk of deterioration of their own health due to RUW than schizophrenia patients (*p* < 0.05). Moreover, they consider themselves more exposed to consequences of RUW that will reduce their well-being in the future (*p* < 0.05).

The results obtained in the negative, positive, and general psychopathology on the PANSS subscales in a sample of schizophrenia patients during base and main measurement were compared. In order to obtain a more accurate picture of changes in symptomatology, the intensity of single 30 PANSS symptoms was also compared. The results are presented in Table 3, however, they only concern significant differences.

As shown above, among patients with schizophrenia, there was significant increase in positive subscale scores in the main measurement (after 24 February 2022) (*p* < 0.05). There were no significant differences in the scores for negative symptoms and general psychopathology. Analyses of single symptoms showed a significant increase in the intensity of delusions, hallucinatory behaviors and conceptual disorganization (all *p* < 0.05). However, no case of deterioration of negative symptoms as well as general psychopathology symptoms was observed in this group of patients after the outbreak of war. Analogical series of *t*-tests in the sample of depressed patients are presented in Table 4.

As Table 4 shows, a significant increase in symptoms was observed in total BDI-II score as well as in the following BDI-II items: loss of pleasure, sadness, pessimism, worthlessness, loss of interest, self-criticalness, and guilt.

In the next step, the indexes of statistically significant change in symptoms (for scales, subscales as well as for single items) were computed using the formula: main measurement minus base measurement. Many analyzes were performed using univariate regression models to identify whether different indexes of symptom change in a group of depressed and schizophrenia patients could be predicted on the basis of exploratory variables (age, sex, marital status, number of children, years of education, years of employment, temperamental traits, and risk perception indexes). Significant independent variables for indexes in schizophrenia patients sample are presented in Table 5.

As shown above, the index of changes in positive symptoms as well as index of changes in delusions, hallucinatory behavior and conceptual disorganization can be predicted based on the level of temperamental perseveration. In addition, emotional reactivity positively influences the indexes of change in positive symptoms and changes in delusional symptoms.

In the group of depressed patients, emotional reactivity had a positive effect on the BDI-II total score change rate as well as indexes of loss of pleasure and interest, sadness, worthlessness, and guilty feelings. Results are presented in Table 6.

Additionally, indexes concerning change in BDI-II total score, loss of pleasure, sadness, and pessimism are positively influenced by the level of perseveration. Finally, subjective concern about one’s own health as well as overall psychological well-being positively affects the BDI-II total score change rate.

## 4. Discussion

The main results indicate that the outbreak of RUW was associated with the intensification of psychiatric symptoms. Moreover, the presented findings suggest the specific patterns of response in patients with different types of psychiatric diagnoses. Depressed patients were more likely to have higher BDI-II total scores and reported significant increases in seven symptoms related to unworthiness and/or guilt, lack of interest and/or pleasure and pessimism. In the group of schizophrenia patients, the reaction to RUW was related with the intensification of positive symptoms. The analysis of individual schizophrenic symptoms showed intensification of delusions, hallucinations, and conceptual disorganization.

The presented study emphasizes the importance of individual differences in temperament as a factor determining the specificity of the extreme stress reaction in patients with severe mental disorders [25]. In both groups, most changes in symptoms were positively predicted by two temperamental traits: emotional reactivity and perseveration. These results are consistent with many data pointing to temperament as the main factor determining an individual’s ability to process external stimuli, hence modifying people’s reactions to an extremely stressful situation and coping strategies [26,27,28]. Emotional reactivity determines the ability to maintain adequate reactions in situations requiring long-term and strongly stimulating activity [37]. So far, the relationship between emotional reactivity and stress-related symptoms has been demonstrated in many situations and in many populations [38,39]. All these studies indicate a positive association between emotional reactivity and intensity of stress-related reactions. Perseveration defines the tendency to continue and/or repeat emotions/reactions/thoughts after the cessation of the stimulus that caused them in a stressful situation [25]. It has been shown to predict persistent forms of depressive symptoms [27], PTSD symptoms [19] and psychotic reactions [39].

It is quite evident that people with mental illnesses are significantly more likely to cope with a war situation in a non-adaptive manner and may report higher levels of psychiatric symptoms in response to this type of extreme stressor. The obtained results are the next step in the process of determining the specificity of this reaction. Different mental disorders may activate specific patterns of response to the threat of war [20]. Depressed people seem to be more conscious of the negative consequences of war than schizophrenia patients, and they assess the subjective risk of health and mental complications as more probable. They react with an aggravation of symptoms related to unworthiness and/or guilt, lack of interest and/or pleasure and pessimism. In other words, war danger seems to blacken the already negative image of depressed self, which in turn increases pain and disorder progression. This form of reaction seems to be consistent with the view that an extremely stressful situation aggravates mental illness and leads to the intensification of symptoms [16,20,24]. Based on the obtained results, it can be concluded that this process is influenced by temperamental traits (see above) and the level of risk perception. The more threatening the reality seems, the greater severity of the symptoms.

The reaction of a schizophrenic patient to the threat of war seems to be limited only to positive symptoms, dependent of temperamental traits and independent of the level of war risk perception. War stress aggravates this group of symptoms [16,20,24]. Additionally, the obtained results may suggest that the increased positive symptomatology may serve as a kind of buffer against an extremely strong stressful situation [20]. Faced with the threat of war, schizophrenia patients invest their cognitive and emotional energy, especially in their internal world, creating a stronger and more consistent system of delusions and hallucinations. This process reduces or masks the sense of danger associated with the real world and minimizes the possibility of deterioration reactive mood, additional decrease in motivation and general aggravation of other symptoms not specific for psychosis. Moreover, an adequate assessment of reality is impossible due to the difficulties in goal-directed thought sequencing and the weakening of the process of targeted thinking (increased conceptual disorganization). This specific buffer against extreme stress may be a kind of defense mechanism developed over the years in patients with schizophrenia. Repetitive childhood traumas, as well as those experienced in adulthood, are particularly prevalent in people with psychosis. So far, existing empirical data has shown that exposure to extremely stressful situations may increase vulnerability to psychotic disorders, with a dose–response relationship in which a greater number of exposures results in stronger risk of psychotic symptoms [40,41]. Aiming to clarify this relationship, researchers have proposed various theses explaining this correlation [42]. Some of them suggest that the positive symptoms of psychosis may in fact be a dissociative phenomenon. In the context of the threat of war, this form of reaction helps to avoid negative thoughts and/or minimize feelings associated with the threat of armed conflict. Unfortunately, this is not a cost-free mechanism; it makes schizophrenic thinking and feeling more unreal and deepens social withdrawal.

The study had several limitations, most of which resulted from procedural constraints imposed by the administration of the psychiatric clinic where the study was conducted. Firstly, it is not known whether the same pattern of results could be found in both study groups during the ongoing psychotherapeutic process. Secondly, it is possible that the effects of the threat of war may be moderated by many other variables, such as personal history of traumatic events and level of social support. Thirdly, only psychiatric symptomatology was measured without analyzing other dimensions of stress response. Moreover, the results obtained were not compared with the patterns of war responses in a control group of healthy people. Fourthly, only two groups of psychiatric patients were analyzed, thus excluding patients with other diagnoses. Finally, there was no focus on the long-term effects of the war, including their potential impact on the further course of mental disorders. Of particular interest would be possible comparisons of Polish patients with mentally ill people from other countries, more physically distant from Ukraine.

## 5. Conclusions

The main results of our study indicate the specific patterns of reactions to war danger for patients with different types of psychiatric diagnosis. Depressed patients reacted with an increase in seven symptoms related to unworthiness and/or guilt, lack of interest, and/or pleasure and pessimism. The response of schizophrenic patients was related only with an increase in positive symptoms. In both groups, most changes in symptoms were positively predicted by two temperamental traits: emotional reactivity and perseveration.

Moreover, subjective concern about one’s own health as well as overall psychological well-being positively affected depression symptoms changes.

To the best of the authors’ knowledge, this is the first study assessing the impact of RUW on psychiatric patients in Europe [5,42,43,44]. It can serve as an important benchmark for the mental health system. The reactions of psychiatric patients to the threat of war may vary. Regardless of the differences, there is a need to raise awareness about this population in terms of the availability of emergency care and ongoing psychiatric care during this extremely dangerous global crisis.

## Figures and Tables

**Table 1:** Differences between groups regarding sociodemographic variables.

Category		Depressed Patients	Schizophrenia Patients	*p*
Age		*M* = 31.12 *SD* = 8.16	*M* = 29.61 *SD* = 10.12	*p* = 0.66
Sex	Women Men	19; 48% 21; 52%	16; 40% 24; 60%	*p* = 0.51
Marital status	Single Married Divorced or separated Widow/Widower	25; 62% 10; 25% 4; 10% 1; 3%	21; 52% 14; 35% 4; 10% 1; 3%	*p* = 0.57
Number of children (mean)		*M* = 1.15 *SD* = 0.83	*M* = 1.02 *SD* = 0.44	*p* = 0.11
Years of education (mean)		*M* = 17.12 *SD* = 5.16	*M* = 11.14 *SD* = 3.13	*p* = 0.02 *
Years of employment (mean)		*M* = 13.11 *SD* = 3.13	*M* = 2.14 *SD* = 3.13	*p* = 0.03 *

*p* for Kruskal–Wallis test for quantitative variables; Chi-squares test for qualitative variables. * *p* < 0.05.

**Table 2:** Differences between groups on RUW risk perception and temperamental traits (before and after exposure of RUW).

Category	Depressed Patients	Schizophrenia Patients	*p*
Temperamental traits			
ER	49 (IQR: 39–56.20)	21 (IQR: 19.25–25)	*p* = 0.01 *
AC	25 (IQR: 14–28.25)	37 IQR:31–54.75)	*p* = 0.03 *
EN	21 (IQR: 28–41.12)	19.30 (IQR: 14–25.90)	*p* = 0.17
BR	35 (IQR: 28–41.12)	38 (IQR: 32.23–44)	*p* = 0.15
SS	26 (IQR: 21–33.30)	41 (IQR: 31.23–47)	*p* = 0.08
PE	51 (IQR: 46–60)	48 (IQR: 40–55)	*p* = 0.19
RT	19 (IQR: 11.30–25)	22 (IQR: 20–25)	*p* = 0.21
RUW risk perception			
HR	7.87 (IQR: 3.3–9.30)	3.31 (IQR: 4.23–7.12)	*p* = 0.04 *
PR	7.11 (IQR: 6.20–9.30)	5.12 (IQR: 2.33–7.33)	*p* = 0.04 *
GWR	5.34 (IQR: 4.00–6.23)	6 (IQR: 3.67–8.00)	*p* = 0.65
I-ER	8.75 (IQR: 7.97–9.40)	7.80 (IQR: 6–8.50)	*p* = 0.12

*p* for Kruskal–Wallis test for quantitative variables. * *p* < 0.05.

**Table 3:** Comparison of results in PANSS scales and single items between the main and base measurement (before and after exposure to RUW).

Category	Base Measurement	Main Measurement	*p*
Main scales score			
Positive symptoms score	30.44 (*SD* = 8.32)	37.52 (*SD* = 8.01)	*p* = 0.01 *
Negative symptoms score	14.70 (*SD* = 5.83)	15.13 (*SD* = 6.06)	*p* = 0.76
General psychopathology score	78.12 (*SD* = 13.33)	80.12 (*SD* = 11.73)	*p* = 0.06
Single items score			
Delusions	*M* = 3.89 (*SD* = 2.13)	*M* = 5.67 (*SD* = 1.76)	*p* = 0.04 *
Hallucinatory behaviors	*M* = 2.14 (*SD* = 1.13)	*M* = 4.58 (*SD* = 2.21)	*p* = 0.03 *
Conceptual disorganization	*M* = 2.13 (*SD* = 0.68)	*M* = 5.11 (*SD* = 2.13)	*p* = 0.03 *

*p* for *t*-test for quantitative variables. * *p* < 0.05.

**Table 4:** Comparison of scores in scales and single items of the BDI-II between the base and main measurement in a group of depressed patients.

Category	Base Measurement	Main Measurement	*p*
Main scales score			
Total BDI-II Score	31.42 (*SD* = 6.57)	36.26 (*SD* = 8.01)	*p* = 0.01 **
Single items score *			
Loss of pleasure	*M* = 1.44 (*SD* = 0.87)	*M* = 2.17 (*SD* = 0.65)	*p* = 0.04 **
Sadness	*M* = 1.44 (*SD* = 0.87)	*M* = 2.11 (*SD* = 0.56)	*p* = 0.03 **
Pessimism	*M* = 2.01 (*SD* = 0.68)	*M* = 2.54 (*SD* = 0.33)	*p* = 0.03 **
Worthlessness	*M* = 1.07 (*SD* = 0.75)	*M* = 2.18 (*SD* = 0.58)	*p* = 0.01 **
Loss of interest	*M* = 1.78 (*SD* = 0.89)	*M* = 2.54 (*SD* = 0.32)	*p* = 0.02 **
Self-criticalness	*M = 1.53 (SD = 0.71)*	*M = 2.11 (SD = 0.54)*	p = 0.04 **
Guilty feelings	*M = 1.13 (SD = 0.76)*	*M = 1.96 (SD = 0.48)*	p = 0.03 **

*p* for *t*-test for quantitative variables. * only significant changes are presented in Table. ** p < 0.05.

**Table 5:** Multiple regression analyses of index of significant changes in symptoms in patients with schizophrenia *.

Category	Positive symptoms Scale		Delusions		Hallucinatory Behaviors		Conceptual Disorganization	
	*β*	*t*	*β*	*t*	*β*	*t*	*β*	*t*
PE	0.29	2.34	0.20	2.43	0.2	2.22	0.21	2.11
ER	0.30	2.7	0.24	2.56	-	-	-	-

* Measured dependent variables: age, sex, years of education, marital status, number of children, years of education, years of employment, temperamental traits (BR, PE, SS, ER, EN, AC and RT) and risk perception indexes (HR, PR, GWR, I-ER). Only significant (*p* < 0.05) independent variables are reported.

**Table 6:** Multiple regression analysis of indexes of significant symptoms change in depressed patients *.

Category	BDI-II Total Score		Loss of Pleasure		Sadness		Pessimism		Worthless-ness		Loss of Interest		Self-Criticalness		Guilty Feelings	
	*β*	*t*	*β*	*t*	*β*	*t*	*β*	*t*	*β*	*t*	*β*	*t*	*β*	*t*	*β*	*t*
Constant	-	2.78	-	2.96	-	2.43	-	3.06	-	3.54	-	4.01	-	-	-	3.03
PE	0.43	2.67	0.20	2.43	0.2	2.22	0.21	2.11	-	-	-	-	-	-	-	-
ER	0.39	2.67	0.2	2.41	0.34	2.19	-	-	0.27	2.54	0.41	2.65	-	-	0.21	2.16
HR	0.24	2.7	-	-	-	-	-	-	-	-	-	-	-	-	-	-
PR	0.36	2.74	-	-	-	-	-	-	-	-	-	-	-	-	-	-

* Measured dependent variables: age, sex, years of education, marital status, number of children, years of education, years of employment, temperamental traits (BR, PE, SS, ER, EN, AC and RT) and risk perception indexes (HR, PR, GWR, I-ER). Only significant (*p* < 0.05) independent variables are reported.

## Data Availability

The data presented in this study are available on request from the corresponding author. The data are not publicly available due to polish restriction concerning the need of protecting medical data of patients.
